# Long-Term Continuation of Outpatient Rehabilitation Improved Lower Limb Muscle Strength and Glycemic Control in a Frail Older Patient With Type 2 Diabetes Mellitus: A Case Report

**DOI:** 10.7759/cureus.87054

**Published:** 2025-06-30

**Authors:** Ryota Shinomiya, Issei Shimizu, Masaaki Nakajima

**Affiliations:** 1 Rehabilitation Department, Tokushima Kensei Hospital, Tokushima, JPN; 2 Department of Human Sciences: Physical Therapy, School of Health Science and Social Welfare, Kibi International University, Takahashi, JPN

**Keywords:** continuous glucose monitoring, exercise therapy, frailty, knee extension force, long-term care insurance system, outpatient rehabilitation, type 2 diabetes mellitus

## Abstract

Type 2 diabetes mellitus (T2DM) is associated with reduced lower limb muscle strength and is closely linked to physical frailty. Frail individuals often face difficulty in engaging in independent exercises, making outpatient rehabilitation (OR) under Japan’s long-term care insurance system a potentially effective intervention. Therefore, in this case, we provided long-term, continued exercise support through OR to an older, frail patient with T2DM. To ensure exercise adherence and prevent acute diabetic complications, we implemented a moderate- or lower-intensity exercise program consisting primarily of aerobic and resistance training, performed twice a week for 80 min per session, over 2 years. Consequently, the knee extension force improved over time. By month 9, the patient had transitioned from frailty to robustness according to the Japanese version of the Cardiovascular Health Study criteria. Regarding glycemic control, hemoglobin A1c (HbA1c) levels remained below baseline throughout the 2-year intervention. Although a temporary increase in HbA1c was observed at month 9, switching from self-monitoring of blood glucose to intermittently scanned continuous glucose monitoring enhanced the patient’s awareness of glycemic fluctuations. Consequently, HbA1c levels were lower in the second year than in the first year. These findings suggest that long-term OR may be beneficial for improving lower limb muscle strength, physical frailty, and glycemic control in older patients with T2DM.

## Introduction

Type 2 diabetes mellitus (T2DM) develops owing to a combination of genetically predisposed reduction in insulin secretion and insulin resistance caused by environmental factors such as high-calorie diets, high-fat intake, and physical inactivity. These factors lead to a relative insulin deficiency [1]. T2DM affects not only glycemic control but also physical function. One such impairment is the faster decline in lower limb muscle strength in patients with T2DM than in individuals without diabetes [2,3]. Concurrent diabetic peripheral neuropathy (DPN) further accelerates the decline in lower limb strength [3].

Nomura et al. conducted a large-scale study on the knee extension force (KEF) in individuals with T2DM in Japan and reported muscle weakness characteristics similar to those described by Andersen et al. [4,5]. Additionally, the aging of the T2DM population is becoming a significant concern. Frailty, which is commonly associated with aging, accelerates the decline in muscle strength, reduces physical activity, and contributes to poor glycemic control in patients with T2DM [6].

Exercise therapy is an important intervention for improving lower limb muscle strength and glycemic control. However, for individuals with muscle weakness or frailty, independent exercise can be difficult, and continuous support is often required. The Japanese Clinical Practice Guidelines for Diabetes 2019 present the target range for glycemic control in older adults. High-intensity exercise is not recommended, especially in those with impaired exercise function or those on insulin therapy. In such cases, less stringent glycemic control is recommended over strict control, considering the risk of hypoglycemia and the overall vulnerability of this population [7].

From the perspective of exercise safety and sustainability, outpatient rehabilitation (OR) covered by Japan’s long-term care insurance system may serve as an effective strategy for improving physical function in patients with T2DM [8]. Continued outpatient rehabilitation also facilitates timely and appropriate diabetes education in cases of poor glycemic control. However, most OR services in Japan are for individuals with cerebrovascular or musculoskeletal disorders, and the proportion of patients with diabetes remains small [9].

Self-monitoring of blood glucose (SMBG) is a standard method of managing glycemic control in patients with T2DM. However, the use of continuous glucose monitoring (CGM) has increased in recent years. CGM allows the real-time tracking of glucose levels and visualizes fluctuations throughout the day. Compared with SMBG, CGM tends to enhance patient awareness of glycemic variability [10]. CGM has also been reported to improve glycemic control in patients with T2DM [11]. Therefore, combining CGM with exercise therapy may enhance the sustainability of therapeutic interventions and provide synergistic benefits for glycemic control.

This case report describes an older, frail patient with T2DM who underwent low-to-moderate intensity exercise therapy with a focus on continuity and safety through OR. The introduction of CGM during a period of deteriorating glycemic control contributed to improvements in exercise capacity, as measured by knee extension force and glycemic regulation. This case provides important insights into the management of frail, older patients with T2DM.

## Case presentation

Patient information

The patient in this case was a woman in her 80s with T2DM (height: 154.0 cm, weight: 36.6 kg, body mass index: 15.4 kg/m^2^, level of care: support needed 2) who used the OR facility covered by Japan’s long-term care insurance system. The complications included DPN, osteoporosis, and a compression fracture of the first lumbar vertebra. Ten months before starting OR, the patient sustained a first lumbar compression fracture and was diagnosed with T2DM at the time of injury. Subsequently, rapid weight loss of approximately 10 kg and poor glycemic control continued over 4 months, resulting in a 1-month educational admission to our hospital. The patient remained hyperglycemic even after discharge and was readmitted to the hospital for glycemic control; however, during this hospitalization, the patient was advised bed rest for COVID-19. Consequently, the patient was discharged from the hospital with residual weight loss, reduced mobility, and poor glycemic control, and started OR at the hospital.

The purpose and objectives of this report and the protection of personal information were explained to the patient in writing and orally, and informed consent was obtained.

Glycemic control status

The pre-intervention hemoglobin A1c (HbA1c) level was 9.1%, and the homeostatic model assessment for beta-cell function value was 2.57%, indicating reduced insulin secretory capacity. The energy intake was 1150 kcal/day, and the energy consumption calculated using the Harris-Benedict formula was 1218 kcal/day, with a Geriatric Nutritional Risk Index (GNRI) of 91.8. Self-monitoring of blood glucose (SMBG) for blood glucose control was performed one to two times/day. Pharmacological treatment consisted of basal-supported oral therapy with sustained-release insulin and oral glucose-lowering drugs. Regarding insulin progression, Tresiba was used at baseline at a dosage of 8 units/day, increased to 10 units/day from month 3, reduced to 8 units/day from month 6, changed to Novolin R at 8 units/day at month 12, and changed again to Tresiba 8 units/day from month 14. The baseline course of oral hypoglycemic medication was Glactive 50 mg once per day, mitiglinide 10 mg twice per day, and glimepiride 1 mg once per day. The Glactive dosage (50 mg) remained unchanged throughout the intervention period. Mitiglinide (10 mg) was changed to three times/day at month 11, and glimepiride (1 mg) was discontinued at month 8.

Motor function indicator

At baseline, the knee extension force (KEF) was 12.4 kgf on the right and 9.7 kgf on the left. The percentage of KEF relative to the body weight (%KEF) was 33.9 on the right and 26.5 on the left. The 30-s chair stand test (CS-30) result was 10 repetitions. The handgrip strength was 11.0 kg in the right hand and 12.0 kg in the left hand. The patients were classified as physically frail based on the Japanese version of the Cardiovascular Health Study criteria (J-CHS) [12], which was developed from the original CHS criteria proposed by Fried et al. [13]. The patient met four of the five criteria: unintended weight loss, weakness, exhaustion, and low physical activity.

The calf circumference was 24.0 cm on the right and 24.5 cm on the left. The 5-m gait speed was 1.21 m/s. The timed up and go (TUG) test time was 19.55 s. The 6-minute walk distance (6 MWD) was 300 m. The Life Space Assessment (LSA) score was 23.5 points, and the average number of steps per day was 1,197.

**Figure 1:**
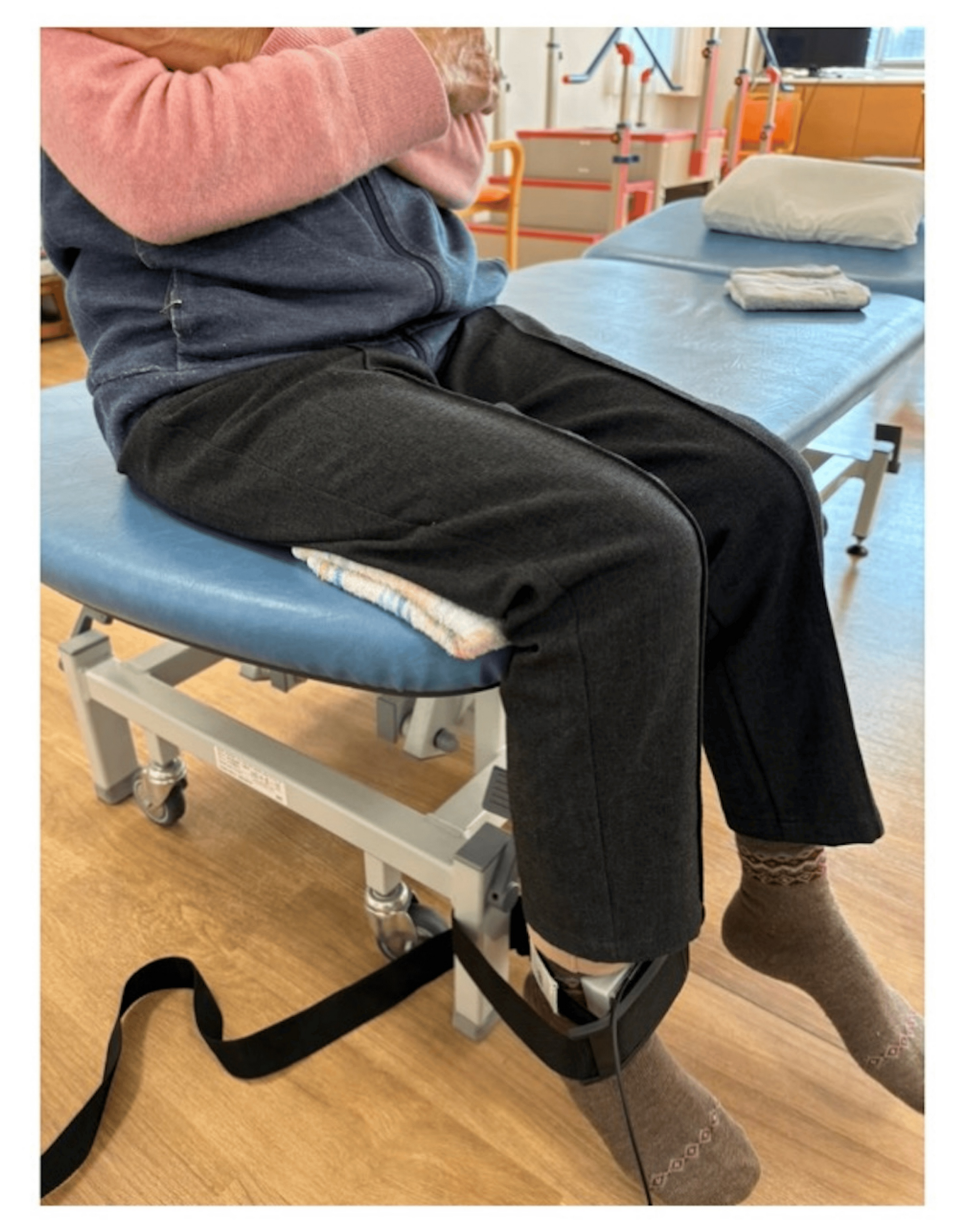
Measurement of the knee extension force (KEF) The position of the limb being assessed was end-sitting, with the knee joint flexed at 90°. A sensor pad was applied to the distal lower leg, and a belt was wrapped around the bed leg to secure it. The patient performed two 5-s maximal isometric knee extension exercises, and the maximum value was adopted.

The KEF was measured using a handheld dynamometer (μTas F-1; Anima Inc., Tokyo, Japan) following the method described by Nomura et al. [4]. The patient was seated with the hips and knees flexed at 90°, and a sensor pad was placed on the distal part of the lower leg. A belt was wrapped around the bed leg to secure the sensor. Two maximal isometric knee extension trials were performed, each lasting 5 s, and the higher value was recorded (Figure 1). Handgrip strength was assessed using a Smedley-type dynamometer (Grip-A; Takei Scientific Instruments Co., Niigata, Japan). The patient was seated with arms hanging naturally at the sides, and two measurements were recorded for each hand. The highest value was used for the analysis.

Intervention

Exercise therapy with OR was continued for 2 years, twice a week, for 80 minutes per session. Notably, only two days of OR were missed during the two-year intervention period, both due to adverse weather conditions.

During the first year of the intervention, the main goal was to improve motor function. Home exercise instructions were provided in addition to exercise therapy at the OR. Exercise therapy consisted of 20 min of individualized exercise therapy (passive static stretching and resistance exercises) by a physical therapist; a self-directed exercise program (half squats, calf raises, balance exercises such as single-leg stance and tandem stance on a balance mat, bicycle ergometer, stepper exercise, hand gripper, stair climbing, hurdle straddle, side walking, backward walking, and shoulder pulley exercises) mainly consisting of aerobic, resistance, and balance exercises for 50 min; automatic limb exercises performed in a sitting position and group exercises consisting of lower limb resistance exercises with weights for 10 min. As part of the self-directed exercise, the exercises were performed while using handrails or parallel bars to ensure safety. The individual exercise program described above was conducted by adding and changing items according to the motor function of the patient. All exercises were performed at below 60% of the heart reserve using the Karvonen method, and below 13 subjective exercise intensity using the Borg scale. For home exercise guidance, the target number of steps was set at 2000-2500 steps/day and managed using a smartphone healthcare application. In addition, simple resistance exercises (calf raises, half squats, abdominal exercises, bridges, and step exercises) and stretching exercises for the gastrocnemius and quadriceps muscles were taught. Considering inadequate energy intake, low nutritional risk, and physical frailty, participants were instructed to increase their food intake with no restriction on energy intake, and their body weight, GNRI, and biochemical parameters were monitored.

Around month 9, when blood glucose control deteriorated, medical care guidance focusing on blood glucose self-management methods was provided. In collaboration with the Department of Internal Medicine and General Practice at the hospital, a FreeStyle Libre (Abbott Diabetes Care, Alameda, CA, USA), an intermittent scanning continuous glucose monitoring (isCGM) system, was introduced to raise awareness of blood glucose fluctuations. The device was replaced with FreeStyle Libre 2 from month 18, both used with a dedicated reader. Feedback was provided on glucose levels, time in range (TIR), and time below range (TBR) obtained using isCGM to improve the awareness of exercise continuation and blood glucose variability.

Assessment task

**Figure 2:**
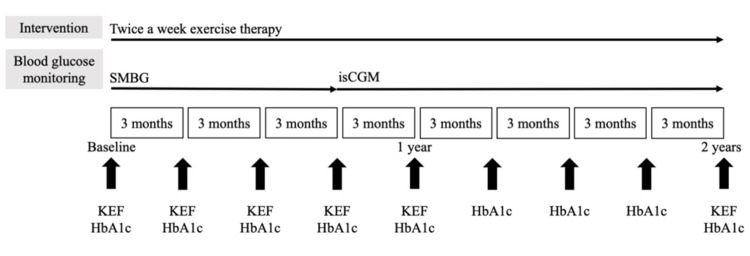
Intervention protocol The exercise therapy protocol was implemented twice weekly, 80 minutes per session, through day rehabilitation for 2 years. Nine months into the intervention, the blood glucose measurement method was changed from self-monitoring blood glucose (SMBG) to intermittent scanning continuous glucose monitoring (isCGM). Motor function, mainly knee extension force (KEF), was assessed every three months for the first year of the intervention and reassessed after two years; glycated hemoglobin (HbA1c) was assessed every three months for two years.

The intervention protocol is illustrated in Figure 2. The main outcomes of motor function were KEF and %KEF. As secondary outcomes, CS-30, grip strength, J-CHS criteria, calf circumference, 5 m gait speed, TUG test time, 6 MWD, LSA score, number of steps, GNRI, and weight were assessed every 3 months during the first year of the intervention to improve motor function. In addition, the same outcomes were reassessed 2 years later.

The main outcome of glycemic control was HbA1c level. Secondary outcomes were renal function and biochemical parameters related to lipid metabolism. In both cases, two-year values were extracted from electronic medical records every three months. After the introduction of isCGM, the TIR, TBR, and number of sensor scans by the reader were analyzed to assess the retention of isCGM and awareness of blood glucose variability.

Outcome of the intervention

Changes in the motor function indices and nutritional status over the two years are shown in Table 1. The KEF, %KEF, and CS-30 improved over time from the start of the intervention. At month 9, physical frailty, according to the J-CHS criteria, improved to robust. After two years, grip strength, body weight, and GNRI also improved and remained robust according to the J-CHS criteria. Calf circumference improved until month 6 and then remained constant at a maintenance level, and the TUG test time showed marked improvement at month 3 of the intervention. The 6 MWD and LSA scores improved steadily over one year and were maintained after two years. By month 9, the patient had more opportunities to eat out and shop with friends and family, and by month 21, she was able to travel by air with her family.

**Table 1:** Changes in motor function and nutritional status mo, months; R, right side; L, left side; KEF, knee extension force; CS-30, 30-s chair stand test; J-CHS, Japanese version of the Cardiovascular Health Study; GNRI, geriatric nutritional risk index; TUG, timed up and go test; 6 MWD, six-min walking distance; LSA, life-space assessment

	Baseline	3 mo	6 mo	9 mo	12 mo	→	24 mo
R-KEF (kgf)	12.4	15.5	16.2	20.8	20.2	→	26.4
L-KEF (kgf)	9.7	14.9	16.1	20.5	19.7	→	23.5
R-%KEF	33.9	39.1	40.5	47.3	46.5	→	58.6
L-%KEF	26.5	37.6	40.3	46.6	45.4	→	52.2
CS-30 (repetitions)	10	12	13	14	17	→	19
R-grip strength (kg)	11.0	16.5	17.0	18.0	19.0	→	22.5
L-grip strength (kg)	12.0	17.0	17.5	19.0	19.5	→	20.0
5 m gait speed (m/s)	1.21	1.18	1.23	1.21	1.29	→	1.26
Body weight (kg)	36.6	39.6	42.8	44.0	43.4	→	45.0
J-CHS criteria	frail	pre-frail	pre-frail	robust	robust	→	robust
GNRI	91.8	91.2	91.5	94.7	94.2	→	94.0
R-calf circumference (cm)	24.0	26.5	28.0	28.5	28.0	→	28.0
L-calf circumference (cm)	24.5	26.5	28.0	28.5	28.0	→	28.0
TUG (s)	19.55	8.88	8.74	8.68	8.41	→	8.45
6 MWD (m)	300	352	345	355	380	→	365
LSA (points)	23.5	49.5	50.5	47.5	62.5	→	59.5
Number of steps (steps/day)	1197	2069	1894	3083	2305	→	2631

Changes in the biochemical parameters and isCGM-related data over two years are shown in Table 2. HbA1c improved up to month 6 but worsened at month 9. At the start of isCGM, the sensor scanned 7 times/day; however, at month 12, when HbA1c was the highest, the sensor scanned 12 times/day. Subsequently, the number of sensor scans ranged from 9 to 21 times per day, and increased awareness of blood glucose variability was observed.

**Table 2:** Changes in biochemical parameters, time in range, time below range, and number of sensor scans mo, months; eGFR, estimated glomerular filtration rate; LDL, low-density lipoprotein; HDL, high-density lipoprotein; TIR, time in range; TBR, time below range

	Baseline	3 mo	6 mo	9 mo	12 mo	15 mo	18 mo	21 mo	24 mo	Reference range
HbA1c (%)	9.1	8.7	7.6	7.8	8.5	8	7.5	7.2	7.8	4.6-6.2
Creatinine (mg/dL)	0.42	0.47	0.51	0.49	0.49	0.58	0.61	0.58	0.58	0.47-0.79
eGFR	105	93	85	89	89	74	70	73	73	≥60
Triglyceride (mg/dL)	102	82	90	70	68	45	77	46	75	30-149
Total cholesterol (mg/dL)	220	230	207	181	192	189	207	175	193	140-219
HDL cholesterol (mg/dL)	61	61	50	47	55	54	51	50	53	≥50
LDL cholesterol (mg/dL)	139	153	139	120	123	126	141	116	125	60-139
TIR (%)	-	-	-	43	32	43	66	52	44	>50
TBR (%)	-	-	-	0	0	0	0	0	0	<1
Number of sensor scans (scans/day)	-	-	-	7	12	13	21	9	13	-

HbA1c levels started to improve from month 3 after the introduction of isCGM. Glycemic control was better in the second year with isCGM compared to the first year. The TBR was 0%, and severe hypoglycemia did not occur during the intervention period.

## Discussion

Summary of results

This report is believed to be the first to demonstrate the usefulness of an intervention combining isCGM with continued exercise support based on a two-year OR for frail older adults with T2DM. In this case report, exercise therapy at a sub-moderate intensity, twice a week, for 80 min per session, was performed in OR for 2 years from the perspective of continuity and safety. Accordingly, the KEF improved over time from frail to robust according to the J-CHS criteria at month 9. Two years later, the KEF further improved and remained robust. In contrast, the HbA1c improved up to month 6 but increased from months 9-12. Thereafter, glycemic control showed an improving trend with increasing isCGM uptake, with overall glycemic control being better in the second year of isCGM use compared to the first year. This report suggests that long-term continuation of OR may contribute to improvements in KEF, physical frailty, and glycemic control in a frail older patient with T2DM.

Improvement of motor function

The KEF and %KEF showed progressive improvement over the first year of the intervention and continued to increase after two years. A similar trend was observed for CS-30. Long-term continuation of OR led to KEF and %KEF values that significantly exceeded the reference values for age-matched patients with T2DM without DPN [4]. Previous reports have indicated that KEF in T2DM patients is associated with regular exercise habits (e.g., exercising for 30 min twice a week for more than month 6) [14], supporting the efficacy of this long-term intervention. In addition to lower limb muscle strength, physical frailty, as assessed using the J-CHS criteria, improved to a robust status by the ninth month. Considering that older patients with T2DM experience a rapid decline in muscle strength [2], these results are clinically significant. Long-term OR may serve as an effective intervention to improve lower limb strength in patients with T2DM.

In contrast, skeletal muscle mass, assessed via calf circumference, increased up to month 6 but was maintained thereafter. Sustained low-intensity exercise exerts a greater influence on muscle strength than on muscle mass, likely through neuromuscular adaptations [15]. In the present case, lower limb strength likely improved because of the reliance on moderate- or lower-intensity exercises. Patients with T2DM often exhibit not only reduced muscle strength but also decreased skeletal muscle mass and increased intramuscular non-contractile tissue [16]. Therefore, interventions targeting skeletal muscle mass are important. In this case, the low baseline calf circumference, reduced creatinine levels (which are dependent on muscle mass), and elevated estimated glomerular filtration rate (eGFR) suggested muscle atrophy. By month 9 of intervention, improvements were noted in calf circumference and creatinine levels, and a decrease in eGFR. Although limited, these findings suggest recovery from muscle atrophy, and the maintenance of these parameters over the subsequent 2 years is clinically meaningful. Furthermore, biochemical parameters related to eGFR, creatinine, and lipid metabolism remained within the normal ranges after two years. Additionally, increases in GNRI and body weight further support the effectiveness of the intervention.

Furthermore, the TUG test, 6 MWD, and LSA scores showed notable improvements beginning in month 3 of the intervention. The minimal detectable change in the TUG test time in older adults requiring care has been reported as 7.17 s [17]. The minimal clinically important difference for the 6 MWD in frail Asian older adults is 17.8 m [18], and the minimal important change in the LSA in community-dwelling older adults is 5 points [19]. These findings support the observation that the present patient’s TUG test, 6 MWD, and LSA scores improved meaningfully within the first year of intervention and that these improvements were maintained over the two years. The overall enhancement in physical function, including KEF, enabled the patient to engage in various real-life activities such as dining out, shopping, and traveling. These outcomes suggest that OR has a positive impact on the patients’ quality of life.

Improvement in glycemic control

The present case involved a patient with insulin-dependent T2DM who required insulin-based pharmacotherapy throughout the intervention period. HbA1c levels remained consistently lower than the baseline values during the intervention. According to a meta-analysis of aerobic exercise, individuals exercising for more than 150 min per week demonstrated greater reductions in HbA1c levels [20]. In this case, the patient engaged in 160 min of exercise therapy per week, comprising multiple components of low to moderate intensity. Although the overall physical activity level was relatively low, the patient achieved the target step count in all months except month 6. When home-based exercises were included, the total exercise duration likely exceeded the prescribed amount. These findings suggest that the continued implementation of exercise therapy contributes to improved glycemic control.

By month 9, the patient’s physical frailty had improved to a robust status, along with general enhancements in physical function. Consequently, the LSA score increased by month 12, and the frequency of dining out increased. These lifestyle changes may have partially contributed to the subsequent increase in HbA1c levels observed after month 9. Another possible factor is seasonal variation, as months 9-12 of the intervention coincided with winter. HbA1c levels have been reported to exhibit seasonal variation, with higher levels typically observed in winter [21]. During the second year of intervention, similar seasonal trends were observed; however, HbA1c levels remained consistently lower than those in the corresponding months of the first year. This suggests that the introduction of isCGM may have contributed to further improvements in glycemic control.

Limitations

The primary limitation of this study is that it is a single-patient case report. Given the heterogeneity of T2DM, which encompasses a wide range of pathophysiological conditions and comorbidities, the interventions described here may not be generalizable to all individuals with T2DM. Nevertheless, this case highlights the fact that exercise support emphasizing continuity through OR led to improvements in lower limb muscle strength, physical frailty, and glycemic control. These findings may provide useful insights for the selection of intervention strategies for older adults with T2DM. Future studies with larger sample sizes are needed to validate these findings.

## Conclusions

We suggest that long-term continuation of low- to moderate-intensity exercise through OR is effective in improving lower limb muscle strength and physical frailty in older adults with T2DM. Additionally, prolonged outpatient support may be a useful strategy for enhancing glycemic management when combined with isCGM. This case suggests that the integration of OR and glucose monitoring may contribute to improvements in both physical function and glycemic control in older adults with T2DM, serving as a foundation for the development of future intervention strategies.
